# Association of *TMEM173/STING1* Gene Variants with Severe COVID-19 Among Fully Vaccinated vs. Non-Vaccinated Individuals

**DOI:** 10.3390/life15081171

**Published:** 2025-07-23

**Authors:** Daniel Vázquez-Coto, Marta García-Clemente, Guillermo M. Albaiceta, Laura Amado, Lorena M. Vega-Prado, Claudia García-Lago, Rebeca Lorca, Juan Gómez, Eliecer Coto

**Affiliations:** 1 Genética Molecular, Hospital Universitario Central de Asturias, 33011 Oviedo, Spain; uo270482@uniovi.es (D.V.-C.);; 2 Neumología, Hospital Universitario Central de Asturias, 33011 Oviedo, Spain; 3 Medicine Department, Universidad de Oviedo, 33011 Oviedo, Spain; rebeca.lorca@sespa.es; 4 Instituto de Investigación Sanitaria del Principado de Asturias, ISPA, 33011 Oviedo, Spain; 5 Functional Biology Department, Universidad de Oviedo, 33011 Oviedo, Spain; 6 Unidad de Cuidados Intensivos Cardiológicos, Hospital Universitario Central de Asturias, 33011 Oviedo, Spain; 7 CIBER-Enfermedades Respiratorias, Instituto de Salud Carlos III, 28029 Madrid, Spain; 8 Instituto Universitario de Oncología del Principado de Asturias (IOUPA), Universidad de Oviedo, 33011 Oviedo, Spain; 9 Cardiología, Hospital Universitario Central de Asturias, 33011 Oviedo, Spain

**Keywords:** COVID-19, RNA vaccines, *STING1*, gene polymorphisms

## Abstract

**Background.** The STING protein is activated by the second messenger cGAMP to promote the innate immune response against infections. Beyond this role, a chronically overactive STING signaling has been described in several disorders. Patients with severe COVID-19 exhibit a hyper-inflammatory response (the cytokine storm) that is in part mediated by the cGAS-STING pathway. Several STING inhibitors may protect from severe COVID-19 by down-regulating several inflammatory cytokines. This pathway has been implicated in the establishment of an optimal antiviral vaccine response. STING agonists as adjuvants improved the IgG titers against the SARS-CoV-2 Spike protein vaccines. **Methods.** We investigated the association between two common functional *STING1/TMEM173* polymorphisms (rs78233829 C>G/p.Gly230Ala and rs1131769C>T/p.His232Arg) and severe COVID-19 requiring hospitalization. A total of 801 non-vaccinated and 105 fully vaccinated (mRNA vaccine) patients, as well as 300 population controls, were genotyped. Frequencies between the groups were statistically compared. **Results.** There were no differences for the *STING1* variant frequencies between non-vaccinated patients and controls. Vaccinated patients showed a significantly higher frequency of rs78233829 C (230Gly) compared to non-vaccinated patients (CC vs. CG + GG; *p* = 0.003; OR = 2.13; 1.29–3.50). The two *STING1* variants were in strong linkage disequilibrium, with the rs78233829 C haplotypes being significantly more common in the vaccinated (*p* = 0.02; OR = 1.66; 95%CI = 1.01–2.55). We also studied the *LTZFL1* rs67959919 G/A polymorphism that was significantly associated with severe COVID-19 (*p* < 0.001; OR = 1.83; 95%CI = 1.28–2.63). However, there were no differences between the non-vaccinated and vaccinated patients for this polymorphism. **Conclusions.** We report a significant association between common functional *STING1* polymorphisms and the risk of developing severe COVID-19 among fully vaccinated patients.

## 1. Introduction

The cGAS-STING pathway is crucial for an appropriate immune response against pathogens. A broad repertoire of dsDNAs, either of foreign and self-origin, can bind to the cGAS protein (cyclic GMP–AMP synthase) in the cytoplasm, activating its catalytic domain and the production of 2′3′ cyclic GMP–AMP (cGAMP) [1]. The cGAMP is a second messenger that binds in the cytoplasm to the STING protein (encoded by the *TMEM173*/STING1 gene) and is able to trigger an innate immune response by enhancing the expression of interferon (IFN) genes. The secreted IFNs promote, in turn, the transcription of several IFN-stimulated genes which are crucial for the antiviral response [2,3,4]. Beyond its critical role in the defense against infections, chronically overactive STING signaling has been found in several pathologies [5,6,7,8,9,10].

SARS-CoV-2, which caused the COVID-19 pandemic, is an RNA virus and is, thus, not directly recognized by the cGAS-STING pathway. However, this pathway has been implicated in the pathogenesis of COVID-19. The STING proteins are able to restrict the infection of RNA viruses by increasing the production of IFN, and SARS-CoV-2 proteins interfere with the STING pathway, impairing the antiviral response [11,12,13,14,15]. The cGAS-STING pathway also modulates pathological processes caused by the presence of cytosolic free mitochondrial DNA (mtDNA), which is commonly seen in viral infected cells [16,17].

Patients with severe COVID-19 exhibit a hyper-inflammatory response with an activation of the NF-kB response, which is mediated by the cGAS-STING pathway [18,19]. This exacerbated immune response is known as a cytokine storm, and is characterized by the recruitment of neutrophils and macrophages that cause the death of lung cells, acute respiratory distress syndrome, and poor clinical outcomes [20]. Moreover, STING inhibitors might protect from severe COVID-19 by down-regulating several inflammatory cytokines [21,22,23].

The STING pathway has been implicated in the establishment of an optimal antiviral response after vaccination [24]. STING agonists induced the priming and differentiation of B-cells, increasing antibody titers after vaccination and extending protection against further viral infection [25]. Moreover, vaccination plus cGAMP or other STING agonists as adjuvants improved the anti-Spike protein IgG titers, increasing protection against further infection [26,27,28,29].

The human *STING1/TMEM173* gene (*chromosome 5*) has several functional single-nucleotide polymorphisms (SNPs). These SNPs have been associated with an impaired IFN response to *L. monocytogenes*, cytokine responses to vaccinia virus stimulation among Europeans, susceptibility to Legionnaires’ disease, disease progression among individuals positive for HIV, several cancer types, and mitochondrial DNA copy number among individuals exposed to polycyclic aromatic hydrocarbons [30,31,32,33,34,35,36,37,38]. The amino acid changes p.R17H (rs11554776), p.G230A (rs78233829), p.R232H (rs1131769), and p.R293Q (rs7380824) are common in Caucasian populations and have been associated with different gene expressions and responses to cyclic dinucleotides [31,32,33,34,35,36,37,38,39]. The rs11554776/rs78233829/rs7380824 SNPs are in complete linkage disequilibrium, and the HAQ protein isoform exhibits reduced intrinsic signaling activity but retains the response to bacterial cyclic dinucleotides [31]. Cells carrying 232^Hys^ showed a significantly reduced expression of type 1 IFNs in response to multiple STING ligands [36]. The two 232 isoforms have different ligand binding characteristics, providing a potential mechanism for different inter-individual responses to viral infection. In particular, the 232^Hys^ could impair STING-mediated innate immunity to poxviruses [40].

Based on the reported evidence that implicated the STING pathway in the pathogenesis of COVID-19 and the antiviral response after vaccination, we hypothesized that common functional *STING1* variants could be associated with the risk of developing severe COVID-19. To address this issue, we studied both vaccinated and non-vaccinated patients who required hospitalization.

## 2. Methods

**Study Cohort.** This study was part of a project to identify the genetic variants associated with the risk of developing severe COVID-19 requiring hospitalization. The study was approved by the Ethical Committee for Medical Research of Asturias (reference CEImPA2022.267, approval date 22/03/2022), and all the participants gave informed consent. The inclusion criteria were the need for hospitalization due to severe pneumonia and positivity for SARS-CoV-2. Patients with immunodeficiency, either primary or secondary to immunosuppressive therapy, were not included in the study. We recruited a total of 906 patients in the period from 1 March 2020 to 31 July 2021, which corresponded to four pandemic waves with hospitalization peaks. The hypertension and dyslipidemia status of each patient was recorded from their clinical history at hospital admission.

A total of 400 patients developed a critical disease that required treatment in the Intensive Care Unit (ICU). The SARS-CoV-2 variant was not determined in all the patients, although the four waves were characterized by the V0/WMV1a/WMV1b (waves 1–2), alpha (wave 3), and delta (wave 4) variants [41]. Waves 1 to 2 and part of the 3rd were prior to the vaccination campaign, which in Spain started on 27 December 2020. Most of the population received an RNA vaccine, and by April 2021, more than 90% of the population aged ≥ 65 years had received the two-dose regimen [42]. Based on the vaccination status, during the 3rd and 4th waves, the patients were classified as non-vaccinated or fully (two doses) vaccinated (Table 1).

A total of 300 individuals from the general population aged 25–85 years (55% male/45% female) were studied to determine the allele and genotype frequencies in the general population. They had been recruited prior to the pandemic’s emergence and no data about their positivity for SARS-CoV-2 was obtained, although none of them had been hospitalized due to COVID-19 during the patient recruitment period.

***TMEM173/STING1* Genotyping.** We used a PCR-RFLP approach to genotype two common SNPs: rs78233829C>G (c.689 G>C, p.Gly230Ala) and rs1131769C>T (c.695 G>A, p.His232Arg). PCR amplifications were digested with *MspI* (rs78233829) or *NlaIII* (rs1131769), and the genotypes from each individual were determined by the size of the digestion fragments after agarose gel electrophoresis. The reliability of the genotyping method was confirmed by Sanger sequencing of PCR fragments with different genotypes.

***LZTFL1* Genotyping.** SNPs at the *LZTFL1* marked the strongest genetic association with severe COVID-19 in genome-wide association studies, with rs17713054 as a probable causative variant [43]. We used a PCR-RFLP approach to study rs67959919 A>G, an SNP in complete linkage disequilibrium with rs17713054. The DNAs were PCR-amplified followed by digestion with *MspI* and agarose gel electrophoresis to visualize the two alleles.

## 3. Statistical Analysis

Statistical analysis was performed to determine the association of the studied variables with the risk of hospitalization due to COVID-19 throughout the pandemic waves. We also compared the critical-ICU vs. non-ICU patients and unvaccinated vs. fully vaccinated. All the patients’ characteristics (age, sex, hypertension, dyslipidemia, vaccination status) were obtained from their clinical history at hospital admission. An age < 65 years was considered as the cut-off value for early-onset COVID-19.

All the data (including the genotypes) were annotated in an Excel file and the statistical analysis was performed via logistic regression with the R-free software (www.r-project.org; version 4.5.1 for windows). Single-linear and multiple logistic regression (R-LGM model) was used to compare the frequency of the study variables between the groups. Haplotype frequencies were calculated online with Cubex (http://apps.biocompute.org.uk/cubex/, accessed 15 December 2024). The difference between the groups was considered statistically significant at a *p*-value < 0.05.

## 4. Results

The main characteristics of the hospitalized COVID-19 patients across the four pandemic waves are summarized in Table 1. The frequency of males, age ≥ 65 years, hypertension, and dyslipidemia was higher in the ICU patients across the different pandemic waves. For the whole non-vaccinated cohort, ICU admission was significantly associated with the four previous variables (*p* < 0.001). Multiple logistic regression showed that these variables were independently associated with the risk of ICU admission, with the next 95% confidence intervals being as follows: male = 1.07–2.11, ≥65 years = 1.01–1.97, hypertension = 1.08–2.20, dyslipidemia = 1.05–2.11.

**Figure 1:**
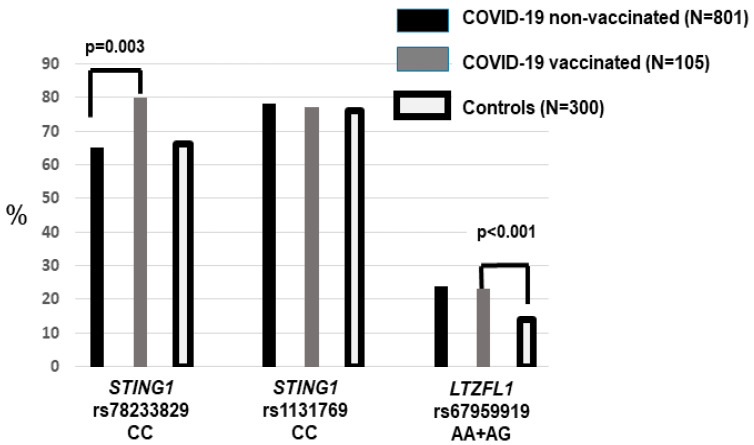
The frequency of the *STING1* and *LZTFL1* risk genotypes in the vaccinated and non-vaccinated COVID-19 patients and the population controls. Hospitalized patients who had been vaccinated showed an increased frequency of rs78233829 CC (*p* = 0.003). The two patient groups (vaccinated and non-vaccinated) showed a higher frequency of *LZTFL1* rs67959919 A.

We determined the allele and genotype frequencies for *STING1* rs78233829 C>G (p.Gly230Ala) and rs1131769 C>T (p.His232Arg) across the hospitalization waves (Table 1). Among the non-vaccinated patients, there were non-significant differences between the ICU and non-ICU patients. Moreover, allele and genotype frequencies did not differ between the entire non-vaccinated and population control groups (Figure 1).

We did not find significant differences for the two *STING1* variants according to sex, hypertension, dyslipidemia, or age < and ≥65 years in the two unvaccinated groups, regardless of ICU and non-ICU status (Table 2). We also performed a multiple logistic regression comparing ICU vs. non-ICU patients with age, sex, hypertension, dyslipidemia, and the two *STING1* variants as covariates. The risk of ICU was significantly increased for males (OR = 1.51, 95%CI = 1.08–2.12), those aged ≥ 65 years (OR = 1.40, 95%CI = 1.01–1.98), those with hypertension (OR = 1.55, 95%CI = 1.08–2.21), and those with dyslipidemia (OR = 1.48, 95%CI = 1.05–2.80), without significant differences for the genotypes.

Among the vaccinated patients, there were only 9 cases who required ICU admission, compared to 96 hospitalized in the ward. Deaths (*n* = 99) were more common among the non-vaccinated (12%), with only 1 death among the 105 vaccinated. Overall, there was a protective effect of vaccination, with a significant reduction in hospitalization and deaths in the group aged ≥ 65 years, in which vaccination was prioritized (Table 1).

The frequency of hypertensives and dyslipidemics was significantly higher (*p* < 0.001) among the vaccinated compared to the non-vaccinated patients. Vaccinated patients also showed a higher frequency of rs78233829 C (230Gly), with a significantly increased frequency of the CC compared to the CG + GG genotype (*p* = 0.003, OR = 2.13, 1.29–3.50). Since these genotypes were not associated with an increased risk of severity (ICU), the higher frequency among the vaccinated who required hospitalization suggested that this effect could be due to poorer immunization among rs78233829 CC patients.

**Figure 2:**
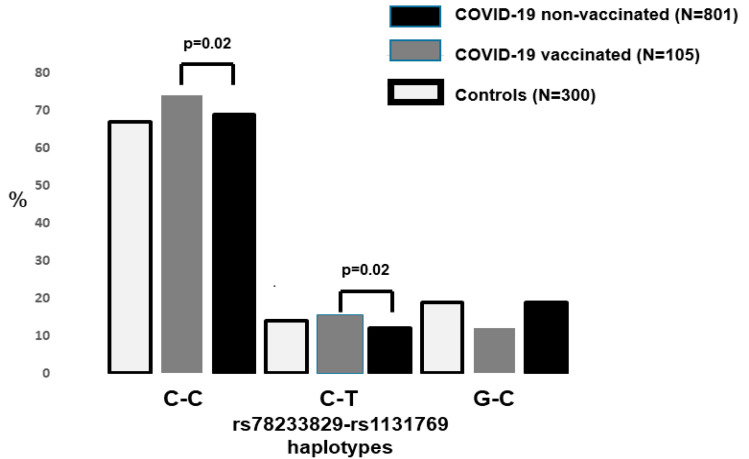
The frequency of the *STING1* risk haplotype in the three study groups. The two rs78233829 C haplotypes were significantly increased (*p* < 0.02) among the hospitalized patients who had been fully vaccinated (*p* = 0.02).

The two *STING1* variants were in strong linkage disequilibrium in both the controls and patients (Figure 2). The two rs78233829 C haplotypes were significantly more common in the vaccinated patients (0.88 vs. 0.81, *p* = 0.02, OR = 1.66, 95%CI = 1.01–2.55).

In reference to the *LZTFL1* rs67959919 polymorphism, there were no differences between ICU and non-ICU or vaccinated vs. non-vaccinated patients (Table 1). Carriers of the rare A allele were significantly more frequent in the group of hospitalized patients compared to the controls (24% vs. 14%, *p* < 0.001, OR = 1.83, 95%CI = 1.28–2.63) (Figure 1). This is in agreement with the widely reported association between *LZTFL1* variants and the risk of developing severe COVID-19. There were no different rs67959919 frequencies between the unvaccinated patients aged < and ≥65 years (Table 2).

## 5. Discussion

The main finding of our study is the significant association between a common functional *STING1* variant and the risk of severe COVID-19 hospitalization among individuals who are fully vaccinated with a SARS-Cov-2 mRNA vaccine. Among the vaccinated patients, we found a significantly increased frequency of the rs78233829 CC genotype. This SNP corresponded to the missense amino acid change p.Gly230Ala in the sting1 protein, with C (230 gly) as the major allele frequency among Europeans. The reported frequency among these populations is in the range C = 0.80–0.86, close to that observed in our controls. We did not find significant differences between vaccinated and non-vaccinated patients for the *STING1* rs1131769 polymorphism (C>T, p.His232Arg). The two *STING1* polymorphisms were in strong linkage disequilibrium in the patients and controls, with the major rs78233829 C-rs1131769 C haplotype showing a significantly increased frequency among the vaccinated patients.

The STING protein has been implicated in the extent of the response to SARS-CoV-2 infection. In particular, patients with severe COVID-19 show an overactive inflammatory response that is in part mediated by the cGAS-STING pathway. This enhanced immune response has features of the cytokine storm that drives the infiltration of neutrophils and macrophages into the lungs, causing acute respiratory distress syndrome and a poor clinical outcome [11,12,13,14,15,18,19]. The fact that some STING inhibitors have exhibited a protective effect also supports the involvement of this pathway in the pathogenesis of critical COVID-19 [21,22,23].

Human cells of individuals carrying the HAQ haplotype showed impaired production of type I IFNs and pro-inflammatory cytokines in response to *L. pneumophila*. Moreover, this haplotype (containing 230^Ala^) was significantly more frequent among patients with Legionnaires’ disease compared to healthy controls, and these *STING1* variants have been associated with HIV progression [32,33]. The amino acid residues 230 and 232 are located in the loop which was predicted to form the c-di-GMP binding pocket of the protein. Previous studies have shown that these and other *STING1* polymorphisms do not alter gene expression and/or stability in HEK293T cells [31]. We did not find significant differences between the *STING1* variants in the patients and controls, nor between patients who required ICU admission or not. These results indicate that, in our population, the two polymorphisms were not associated with the risk of developing severe COVID-19 among the non-vaccinated.

The cGAS-STING pathway has been recognized as an important player in the establishment of an optimal vaccination response [21,22,23]. Several STING agonists, including cGAMP, have been proposed as adjuvants to increase the IgG titers against the S-protein, which would enhance the protection against further SARS-CoV-2 infection [26,27,29,44,45]. The effect of common *STING1* variants on the response to immune challenge has been addressed by some authors. Li et al. determined whether several common haplogroups could affect the recognition of exogenous cyclic dinucleotides. In the absence of exogenous ligands, the *STING1* haplotypes showed a modest difference in the stimulation of the NF-κB and IFN-β pathways [22]. However, in the presence of c-di-GMP, the authors found significant differences between the HAQ and RGR (wild-type) haplotypes. Also, the efficacy of a pneumococcal polysaccharide vaccine could depend on the TMEM173/STING1 pathway and differ between the *STING1* haplotypes [46]. In our study, the HAQ haplotype (defined as rs67959919 G, 230^Ala^) was significantly reduced among the vaccinated patients who developed severe COVID-19, pointing to an impaired protection in homozygotes for the common wild-type RGR haplotype (230^Gly^).

We also determined the effect of the common *LZTFL1* risk variant on the risk of severe disease among the vaccinated. *LZTFL1* variants have been widely identified as a genetic risk factor for severe COVID-19 via a mechanism that could involve enhanced signaling of the epithelial–mesenchymal transition in pulmonary epithelial cells [42]. The risk rs67959919 genotypes were significantly increased in patients across the different hospitalization waves, without differences between the vaccinated and non-vaccinated. Thus, this variant would contribute to the risk of severe COVID-19 independently of the vaccination status.

Finally, our study has several limitations. First, since the *STING1* genotypes were not associated with a higher risk of hospitalization or ICU admission, the higher frequency of rs78233829 CC among the vaccinated who required hospitalization suggested that this effect could be due to poorer immunization among rs78233829 CC individuals. However, the number of vaccinated patients was low, meaning replication is required in large cohorts from different populations. Second, although we studied patients from four pandemic waves characterized by different SARS-CoV-2 strains, the variant was not identified in all of them and a precise effect of the *STING1* polymorphisms depending on the variant could not be established. Also, we determined the short-time protective effect of two vaccine doses and did not analyze a long-term effect of vaccination. It is also necessary to determine whether the rs78233829 C risk allele was associated with reduced IgG titers after vaccination, which could explain the increased risk for severe COVID-19 among the vaccinated with this variant.

In conclusion, we reported a significant association of common *STING1* SNPs/haplotypes with the risk of hospitalization due to COVID-19 among individuals who were fully vaccinated. These variants were not associated with the risk of severe COVID-19 among the non-vaccinated. Thus, our study suggests that common functional *STING1* polymorphisms could be associated with an improved vaccination response against SARS-CoV-2. However, the study was based on a limited number of cases and from a single population and would, thus, require validation in additional studies.

## Figures and Tables

**Table 1:** Main characteristics of the COVID-19 patients and population controls.

	1st–2nd WaveNon-Vaccinated		3rd–4th WaveNon-Vaccinated		3rd–4th WaveVaccinated		Controls
	Non-ICU	ICU	Non-ICU	ICU	Non-ICU	ICU	
	*n* = 225	*n* = 216	*n* = 185	*n* = 175	*n* = 96	*n* = 9	*n* = 300
Male	133 (59%)	140 (65%)	113 (61%)	128 (73%)	60 (63%)	7 (78%)	165 (55%)
Female	92 (41%)	76 (35%)	72 (39%)	47 (27%)	36 (37%)	2 (22%)	135 (45%)
<65 years	143 (64%)	94 (44%)	145 (78%)	102 (58%)	20 (21%)	2 (22%)	160 (53%)
≥65 years	82 (36%)	122 (56%)	40 (22%)	73 (42%)	80 (79%)	7 (78%)	140 (47%)
Death	5 (2%)	59 (27%)	2 (2%)	32 (18%)	0	1 (11%)	-
Hypertensives	85 (38%)	127 (59%)	59 (32%)	95 (54%)	60 (63%)	6 (67%)	-
Dyslipidemia	68 (30%)	98 (45%)	56 (30%)	83 (48%)	49 (51%)	5 (56%)	-
*STING1* rs78233829 C/G							
GG	4 (2%)	10 (4%)	9 (5%)	9 (5%)	4 (4%)	0	11 (3%)
CG	72 (32%)	64 (30%)	56 (30%)	54 (31%)	15 (16%)	2 (22%)	92 (31%)
CC	149 (66%)	142 (66%)	120 (65%)	112 (64%)	77 (80%)	7 (78%)	197 (66%)
MAF G	0.18	0.19	0.20	0.21	0.12	0.11	0.19
*STING1* rs1131769 C/T							
TT	4 (2%)	4 (2%)	2 (1%)	3 (2%)	4 (5%)	0	11 (4%)
CT	57 (25%)	38 (18%)	40 (22%)	30 (17%)	18 (19%)	1 (11%)	62 (21%)
CC	164 (73%)	174 (81%)	143 (77%)	142 (81%)	73 (76%)	8 (89%)	227 (76%)
MAF T	0.14	0.11	0.12	0.10	0.14	0.06	0.14
*LZTFL1* rs67959919 A/G							
AA	3 (1%)	2 (1%)	4 (2%)	4 (3%)	1 (1%)	0	4 (1%)
AG	44 (20%)	54 (25%)	37 (20%)	40 (23%)	21 (22%)	2 (22%)	39 (13%)
GG	178 (77%)	160 (74%)	144 (78%)	131 (75%)	74 (77%)	7 (78%)	157 (86%)
MAF A	0.11	0.13	0.12	0.14	0.12	0.11	0.12

**Table 2:** Distribution of the three SNPs’ frequencies in non-vaccinated patients aged < and ≥65 years old.

	<65 YEARS			≥65 YEARS		
	ICU *n* = 196	NON-ICU *n* = 288	*p*-Value *	ICU *n* = 195	NON-ICU *n* = 122	*p*-Value *
rs78233829						
GG	130 (66%)	189 (65%)	ns	124 (64%)	80 (66%)	
CG	55 (28%)	89 (31%)		63 (32%)	39 (32%)	ns
CC	11 (6%)	10 (4%)		8 (4%)	3 (2%)	
MAF G	0.20	0.19		0.20	0.19	
rs1131769						
CC	153 (78%)	217 (75%)		163 (84%)	90 (74%)	
CT	40 (20%)	65 (23%)	ns	28 (14%)	32 (26%)	ns
TT	3 (2%)	6 (2%)		4 (2%)	0	
MAF T	0.12	0.13		0.10	0.13	
rs67959919						
AA	6 (3%)	5 (2%)	ns	0	2 (2%)	ns
AG	49 (25%)	58 (20%)		45 (23%)	23 (19%)	
GG	141 (72%)	225 (78%)		150 (77%)	97 (80%)	
MAF A	0.16	0.12		0.12	0.11	

* Minor allele carriers vs. most common allele homozygotes. ns: non-significant

## Data Availability

The materials and raw data described in the manuscript will be freely available to any researcher without breaching participant’s confidentiality. To facilitate the revision of the results by other researchers, a file with the patient’s data is available as an excel file upon request to the corresponding author.
